# β1 integrin mediates an alternative survival pathway in breast cancer cells resistant to lapatinib

**DOI:** 10.1186/bcr2936

**Published:** 2011-08-31

**Authors:** Catherine Huang, Catherine C Park, Susan G Hilsenbeck, Robin Ward, Mothaffar F Rimawi, Yen-chao Wang, Jiang Shou, Mina J Bissell, C Kent Osborne, Rachel Schiff

**Affiliations:** 1 Lester and Sue Smith Breast Center, Baylor College of Medicine, One Baylor Plaza, Houston, TX 77054, USA; 2 Department of Radiation Oncology, University of California San Francisco, 1600 Divisadero Street, MZ Bldg R, San Francisco, CA 94143, USA; 3 Department of Cancer & DNA Damage Responses, Lawrence Berkeley National Laboratories, One Cyclotron Road, Berkeley, CA 94720, USA

## Abstract

**Introduction:**

The overexpression of human epidermal growth factor receptor (HER)-2 in 20% of human breast cancers and its association with aggressive growth has led to widespread use of HER2-targeted therapies, such as trastuzumab (T) and lapatinib (L). Despite the success of these drugs, their efficacy is limited in patients whose tumors demonstrate *de novo *or acquired resistance to treatment. The β1 integrin resides on the membrane of the breast cancer cell, activating several elements of breast tumor progression including proliferation and survival.

**Methods:**

We developed a panel of HER2-overexpressing cell lines resistant to L, T, and the potent LT combination through long-term exposure and validated these models in 3D culture. Parental and L/T/LT-resistant cells were subject to HER2 and β1 integrin inhibitors in 3D and monitored for 12 days, followed by quantification of colony number. Parallel experiments were conducted where cells were either stained for Ki-67 and Terminal deoxynucleotidyl transferase dUTP nick end labeling (TUNEL) or harvested for protein and analyzed by immunoblot. Results were subjected to statistical testing using analysis of variance and linear contrasts, followed by adjustment with the Sidak method.

**Results:**

Using multiple cell lines including BT474 and HCC1954, we reveal that in L and LT resistance, where phosphorylation of EGFR/HER1, HER2, and HER3 are strongly inhibited, kinases downstream of β1 integrin--including focal adhesion kinase (FAK) and Src--are up-regulated. Blockade of β1 by the antibody AIIB2 abrogates this up-regulation and functionally achieves significant growth inhibition of L and LT resistant cells in 3D, without dramatically affecting the parental cells. SiRNA against β1 as well as pharmacologic inhibition of FAK achieve the same growth inhibitory effect. In contrast, trastuzumab-resistant cells, which retain high levels of phosphorylated EGFR/HER1, HER2, and HER3, are only modestly growth-inhibited by AIIB2.

**Conclusions:**

Our data suggest that HER2 activity, which is suppressed in resistance involving L but not T alone, dictates whether β1 mediates an alternative pathway driving resistance. Our findings justify clinical studies investigating the inhibition of β1 or its downstream signaling moieties as strategies to overcome acquired L and LT resistance.

## Introduction

The HER signaling pathway is one of the most studied and prominent drivers of human breast cancer progression. Aberrant overexpression, activation, and dimerization of the individual members of the HER family--comprised of EGFR (Epidermal Growth Factor Receptor 1)/HER1, HER2, HER3, and HER4--contribute both to aggressive tumor growth and poor patient prognosis [1]. Amidst the complexity of the HER signaling network, HER2 has received a great deal of attention due to its frequent overexpression in tumors and its status as the preferred dimerization partner of the family [2].

HER2 is amplified and/or overexpressed in about 20% of human breast cancers and is independently associated with reduced disease-free and overall survival. Two FDA-approved drugs for the treatment of HER2-overexpressing tumors are the monoclonal antibody trastuzumab, and the EGFR/HER2 tyrosine kinase inhibitor lapatinib. Each drug is effective in inducing tumor regression in some patients with metastatic disease, but remissions are temporary since resistance commonly develops [3-9]. Clinical trials are currently investigating the administration of lapatinib and trastuzumab together (LT) [8-10], which has been shown pre-clinically by our laboratory [11] and others [12,13] to induce prolonged regression in breast cancer xenografts by more completely blocking downstream signals generated by various homo- and hetero-dimers of the HER family. Even this potent treatment strategy, however, gives way to resistance in many tumors. It is clear that the identification of alternative molecular pathways driving resistant growth would have important therapeutic implications.

The β1 integrin subunit is one member of a large family of receptors that mediate the interaction between cytoskeletal elements and the extracellular matrix [14]. Each integrin is a heterodimer composed of one of 18 possible α subunits together with 1 of 8 β subunits. In response to laminin or fibronectin [15-21], β1 as a mechanoreceptor is a critical mediator of breast cancer initiation and progression [20,22-24], both through its association with the HER pathway [25] and signal propagation through its downstream kinases FAK and Src [26-29]. In addition, β1 has been linked to therapeutic resistance in multiple cancer types [30-32], its overexpression has been associated with poor overall survival in patients with early-stage breast cancer [33], and it can serve as a predictive indicator for patients with intrinsic resistance to trastuzumab [34].

Using an array of HER2-overexpressing cell lines [14,35] developed to acquire resistance (Res) to lapatinib (L), trastuzumab (T), or both (LT) [36], we now report the critical role of β1 integrin as an alternative pathway in L- and LT resistance. We demonstrate that L- and LTRes cells maintain strong inhibition of HER2 as well as EGFR and HER3. However, in resistant cells phosphorylation of β1 downstream kinases FAK and Src is markedly upregulated, and this is inhibited by the β1 antibody AIIB2. We also show that β1 blockade by either AIIB2 or siRNA, as well as by a FAK inhibitor, significantly inhibits L- and LTRes cell growth in 3D. Parental and TRes cells, on the other hand, which retain high levels of phosphorylated EGFR, HER2, and HER3, fail to up-regulate the β1 pathway, and respond to AIIB2 with only modest growth inhibition, suggesting that these cells rely less on the β1 pathway than the HER pathway for growth and resistance, in contrast to LRes and LTRes cells. Most importantly parental and TRes cells in our 3D models respond to L, indicating that their growth is due to the HER pathway. Altogether our results indicate that when HER2 is inhibited, as it is in L- and LTRes breast cancer cells, β1 signaling can operate as an alternative driver of growth.

## Materials and methods

### Reagents and cell culture

The human breast cancer cell line BT474 was purchased from American Type Culture Collection (ATCC, Manassas, VA, USA) and maintained as previously described [37]. AU565, HCC202, and HCC1954 cell lines were generously supplied by Dr. J. Gray (Berkeley, CA, USA) and grown as in [35]. Cell lines were not re-authenticated upon receipt. Three-dimensional (3D) cultures were plated as in [20] on top of growth factor-reduced lrECM (Trevigen, Inc., Gaithersburg, MD, USA) at a density of 3 to 6,000 cells per well of an eight-well chamberslide (LabTek, Milwaukee, WI, USA), with all relevant inhibitors added on Day 0. Media containing the inhibitors and 5% lrECM were changed every 3 days and maintained for 5 to 12 days. The β1 inhibitory antibody AIIB2 (Aragen Bioscience, Morgan Hill, CA, USA, used at 0.24 mg/ml), FAK inhibitor PF 573228 (Tocris Bioscience, Bristol, UK, used at 1 μM), lapatinib (GlaxoSmithKline, Research Triangle Park, NC, USA, used at 1 μM), and trastuzumab (Genentech, San Francisco, CA, USA, used at 50 μg/ml) were used. Control vehicles were rat IgG (Thermo Fisher Scientific, Waltham, Massachusetts, USA) and dimethyl sulfoxide (DMSO). Antibodies used include phosphorylated (p) HER2 (Y1248, Millipore, Billerica, Massachusetts, USA); pHER2 (y877), pEGFR (Y1068), EGFR, pHER3 (Y1289), HER3, pAKT (S473), AKT, pSrc (Y416), and β-actin (all from Cell Signaling Technology, Danvers, Massachusetts, USA); β1 and FAK (BD Biosciences, Franklin Lakes, New Jersey, USA); pFAK (Y861, Biosource, Chevy Chase, Maryland, USA); and Src and HER2 (Santa Cruz Biotechnology, Santa Cruz, CA, USA).

### Establishment of resistant cell lines

Resistant cell lines were developed by first exposing cells to a full dose of lapatinib (1 μM) and/or trastuzumab (50 μg/ml) to assess the *de novo *resistance of each model. Drug-sensitive cell lines were subsequently cultured for 3 to 12 months in respective media supplemented with increasing concentrations of lapatinib (0.1 to 1 μM), trastuzumab (1 to 50 μg/ml), or both, as cells developed resistance to each dosage. Resistance at each dose was assessed by comparing the growth of each resistant derivative to their parental counterparts. All resistant cells were passaged along the same schedule, twice a week, with resistant cells being split at equal or higher ratios than the parental. HCC1954 cells are *de novo *resistant to trastuzumab and were cultured in the continuous presence of the inhibitor both before and during experimentation.

### siRNA in 2D/3D

Cells were seeded in six-well plates at 60% confluency and subjected to two consecutive 24-hour rounds of siRNA transfection, using Hs_ITGB1_5 oligos (Qiagen, Hilden, Germany) and the Mirus Trans-IT TKO transfection reagent (Madison, WI, USA). Cells were then trypsinized and plated on top of lrECM as above. Assay medium containing 5% lrECM, Mirus reagent, siRNA-β1, and relevant drugs was supplied to the cells and changed every three days. Cultures were propagated for 10 days and imaged. A second siRNA was utilized for data in Additional file 1 (catalog s7576, Applied Biosystems, Carlsbad, CA, USA).

### Protein extraction and immunoblot

Protein extractions from 2D cultures were performed as in [35]. All panels shown were run on the same gel per cell model or, when working with overlapping markers, run on two gels in parallel. All experiments were repeated at least three times and representative results are shown.

From 3D cultures seeded in 35 mm dishes for at least five days, wells were washed twice with cold PBS, then replaced with PBS-EDTA (containing 5 mM EDTA, 1 mM 0.2 M Na3VO4, 1.5 mM 0.25 M NaF, and a cocktail of protease inhibitors (Roche, Basel, Switzerland)) for 15 minutes on ice. Cultures and lrECM were then separated from the dish using a cell lifter, and contents were transferred to a conical tube for an additional 30 minutes on ice to allow full dissolution of the lrECM. Cells were then centrifuged for five minutes at 800 rpms, the supernatant aspirated, and the pellet resuspended in lysis buffer. Protein was extracted as above and subjected to Western blot analysis.

### Apoptosis and proliferation assays

Apoptosis (TUNEL assay) and proliferation were assayed in-well of an eight-well chamberslide as in [20] with the following modifications: cells were fixed in 4% paraformaldehyde and Ki-67 was obtained from Vector Labs, Burlingame, CA, USA, and detected by Alexa Fluor 568 goat anti-rabbit IgG secondary antibody (Molecular Probes/Invitrogen). All slides were imaged using a Leica EL6000 inverted laser scanning confocal microscope.

### Quantification of 3D cultures

All experiments were conducted in triplicate and repeated in three independent studies. Phase contrast images of the cultures in eight-well chamberslides were taken with a 4x objective lens, then processed and quantified by measuring colony size. A conversion factor of 1.8755 μm/pixel for the 4x objective was determined and utilized to acquire numerical measurements for each colony imaged, with the cutoff of 50 μm set as the defining value for a colony. At least nine wells per treatment group were quantified per value reported, from three independent experiments.

### Statistical considerations

*In vitro *experiments to compare numbers of colonies formed, proliferation, and apoptosis were run in triplicate and repeated at least three times. The experiment sets had factorial designs and were analyzed using analysis of variance, which allows global analysis of all experiments in the set. All data were first transformed, by taking logarithms, in order to stabilize variances and because differential effects detected by ANOVA on the log scale can be expressed as fold changes on the raw scale, and have a natural biologic interpretation. Each "experiment" was considered a categorical blocking factor, cell line (if different cell lines were used) was a categorical factor (that is, HCC1954 and HCC1954 LTRes), and treatment (that is, IgG vs AIIB2) was a categorical factor, yielding three-way or two-way factorial designs. Analyses included "experiment" as a main effect only and included main effects and interaction between cell line and treatment, where appropriate. Specific comparisons (for example, to examine IgG vs AIIB2 within HCC1954, after adjusting for experiment) were made using linear contrasts. *P*-values for comparisons of treatments were adjusted by the Sidak method to account for multiple comparisons. For purposes of plotting, geometric means and 95% confidence intervals were calculated by back-transforming (that is, exponentiating) model-estimated group means and 95% confidence limits. Plots show data from three repeated experiments, executed in triplicate, combined.

## Results

### The β1 integrin's downstream kinases FAK and Src are activated upon acquisition of resistance to lapatinib-containing HER2-targeted therapies

**Figure 1:**
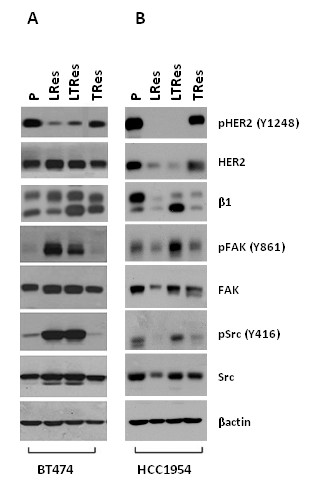
**Phosphorylated levels of β1 downstream kinases are increased upon acquisition of resistance to lapatinib (L)**. (**A**) Parental (P) BT474 and (**B**) HCC1954 cells resistant to lapatinib (LRes), trastuzumab (TRes), and combination (LTRes) treatment strategies were developed by long-term exposure in 2D. Protein extracts were probed for β1, pHER2, pFAK, and pSrc, as well as totals.

We developed a panel of HER2-overexpressing cell lines resistant to lapatinib (LRes), trastuzumab (TRes), and the LT combination (LTRes) through long-term culturing in 2D. Immunoblot analysis of these acquired resistant cell lines revealed that phosphorylated (p) HER2 levels were mostly reduced in both LRes and LTRes BT474 (Figure 1A) and HCC1954 (Figure 1B) cells in comparison to the untreated parental, but retained high expression in acquired (BT474) or *de novo *(HCC1954) TRes cells. In BT474 LRes, BT474 LTRes, and HCC1954 LTRes cells, where HER2 phosphorylation was mostly inhibited, we found marked increases in levels of the β1 integrin downstream kinases pFAK (Y861) and pSrc (Y416) (Figure 1). Interestingly, HCC1954 LRes cells, which express low levels of all markers examined, did not grow in lrECM. Additional cell lines, AU565 and HCC202, yielded similar results (Additional file 2). These data led us to hypothesize that persistent inhibition of pHER2 in LRes and LTRes (but not TRes) can lead to activation of FAK and Src as an escape mechanism to circumvent HER2 blockade, and that targeting their shared upstream protein, the β1 integrin (β1), could potentially circumvent the consequent resistance.

### β1 inhibition overcomes L resistance in ER+, HER2-amplified BT474 cells

**Figure 2:**
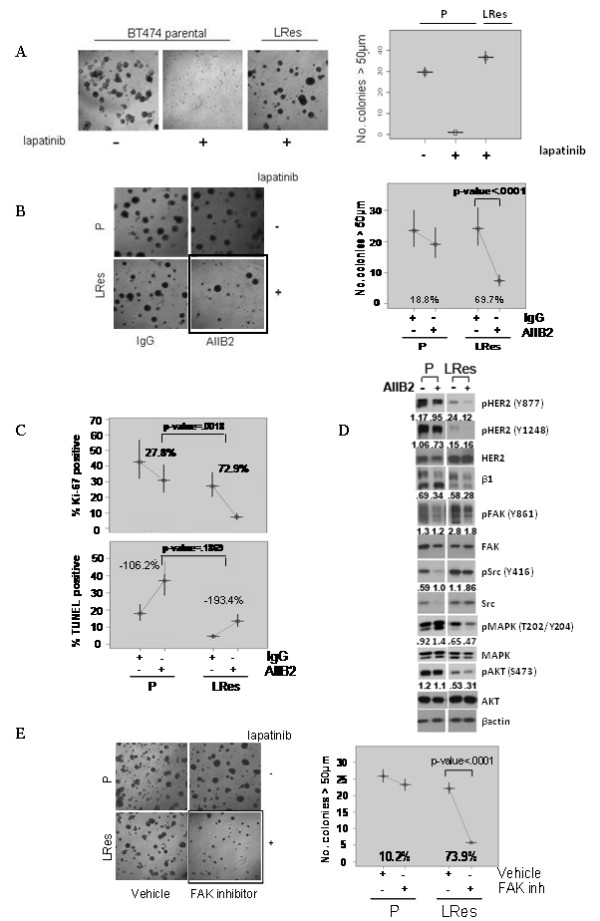
**β1 blockade overcomes lapatinib resistance in BT474 cells in 3D culture**. (**A**) Parental and LRes cells were plated on lrECM and treated ± lapatinib. (**B**) The β1 inhibitory antibody AIIB2--or IgG control--was applied to parental and LRes cells on Day 0 of plating on lrECM and allowed to grow for 12 days. (**C**) Parental and LRes cells were plated in lrECM with appropriate inhibitors, allowed to grow for five days, stained with Ki67 antigen or TUNEL labeling, and imaged. (**D**) 3D cultures of BT474 parental and LRes cells treated with AIIB2 or IgG were allowed to propagate for five days, then harvested for protein and immunoblotting. Densitometry measurements were normalized to total levels--except β1, which was normalized to β-actin--and are representative of three independent experiments. (**E**) The FAK inhibitor PF 573228 was applied to parental and LRes cells on Day 0 of plating on lrECM and cultures were allowed to grow for 12 days.

We next tested β1's role in resistance by blocking its activity using either the inhibitory antibody AIIB2, which binds the extracellular portion of the β1 integrin to inhibit signaling, or β1-specific siRNA in laminin-rich extracellular matrix (lrECM) 3D culture. We used 3D culture because of its ability to recapitulate β1's *in vivo *function, which is to mediate the communication between extracellular signals and intracellular kinases. To first validate our resistant models in 3D culture, BT474 parental and LRes cells were propagated on lrECM and assayed for growth. As expected, parental cells displayed robust growth characteristic of tumorigenic cells in 3D, but they were profoundly growth-inhibited by treatment with lapatinib. LRes cells, on the other hand, grew aggressively in its presence (Figure 2A and Table 1).

**Table 1:** Percent growth inhibition of cells in response to HER-targeted therapies

Cell line	Drug →	Lapatinib	Trastuzumab	LT
	Derivative	% Inhibition (95% CI)	% Inhibition (95% CI)	% Inhibition (95% CI)
**BT474**	**Parental**	100.0%	72.1% (67.6%, 76.0%)	100.0%
	**Resistant**	5.7% (-0.3%, 11.4%)	11.4% (5.8%, 16.8%)	13.7% (8.6%, 18.6%)
**HCC1954**	**Parental**	---	7.4% (3.8%, 10.9%)	100.0%
	**Resistant**	---	5.4% (1.4%, 9.2%)	37.8% (32.5%, 42.7%)

An analysis of variance was performed to estimate the effect of treatment on each cell model. The estimated treatment effect and associated standard error were used to compute estimates and 95% confidence intervals, which were then back-transformed and subtracted from 100% to give the "% inhibition."

We then subjected parental and LRes cells to AIIB2 treatment and found it to be highly effective in inhibiting colony growth in LRes cells (69.7% colony growth inhibition, *P*-value <.0001), while only modestly and nonsignificantly affecting parental cells (18.8% colony growth inhibition, *P*-value = .2498) (Figure 2B). Examination of an additional cell line, AU565 LRes, produced similar results (Additional file 3A).

To investigate the nature of the growth inhibition by AIIB2, we assessed the proliferative (Ki-67) and apoptotic (TUNEL) indices of parental and LRes BT474 cells (Figure 2D). AIIB2 inhibited proliferation only modestly and nonsignificantly in parental cells, whereas proliferation was significantly inhibited in LRes cells (72.9%, *P *<0.001). The degree of inhibition was significantly greater in LRes cells compared to parental cells (*P *= 0.0018). In contrast, both parental and LRes cells underwent increased apoptosis with AIIB2 treatment (106%, *P *<0.001; 193%, *P *<0.001, respectively), but the degree of increase was not significantly different between the cell lines (*P *= 0.1869, Figure 2C). Collectively these data suggest that AIIB2 elicits its differential growth inhibitory effects on LRes BT474 cells primarily by inhibiting proliferation.

### β1 downstream signaling is inhibited by AIIB2 and is critical for the lapatinib resistant phenotype

We then determined how β1 inhibition by AIIB2 could alter downstream signaling in LRes BT474 3D cultures (Figure 2D). Confirming data from Figure 1, we found that in comparison to the parental, LRes cells exhibited dramatically less pHER2 at two independent phosphorylation sites (Y877 and Y1248), while exhibiting dramatic increases in both pFAK and pSrc. The upregulation of pFAK and pSrc in LRes cells was abrogated by treatment with AIIB2. The top band of the β1 doublet, which we believe represents posttranslational modification, was also reduced by AIIB2. Levels of pMAPK and pAKT, which reside downstream of both β1 integrin and HER receptor signaling, were also altered. pMAPK (T202/Y204) expression was decreased in the LRes line and, importantly, further diminished with AIIB2 treatment. pAKT (S473) expression showed a similar trend (Figure 2D). These experiments, which were confirmed with AU565 cells in Additional file 3B, suggest that β1 integrin and its downstream moieties FAK and Src, as well as MAPK and AKT, are important in BT474 LRes cells, and that neutralizing this pathway may provide a functional basis for the ability of β1 blockade to overcome lapatinib resistance. Interestingly, both phospho- and total Src levels were reduced to undetectable levels in parental cells treated with AIIB2.

To further confirm the importance of the β1 downstream signaling pathway in LRes cells, we utilized the FAK inhibitor PF 573228 [38]. Results mirrored our findings with AIIB2. Parental cells responded to FAK inhibition in 3D with a small, nonsignificant reduction in colony growth, while LRes cells were significantly more sensitive, exhibiting a 73.9% (*P *<0.0001) reduction in colony growth (Figure 2E). These data suggest that β1 and FAK are far more important for the LRes phenotype than for growth of parental cells and, thus, their blockade elicits more striking inhibition of LRes cells.

### TRes BT474 cells are inhibited by lapatinib

**Figure 3:**
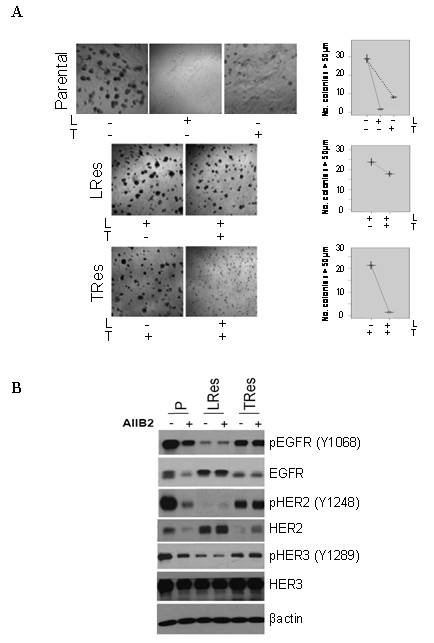
**HER2 and the β1 pathway play alternate roles in resistance to lapatinib-containing regimens, in comparison to trastuzumab**. (**A**) Parental, LRes, and TRes cells were plated on lrECM in the presence of lapatinib and/or trastuzumab and assayed for response. (**B**) 3D cultures of parental, LRes, and TRes BT474 cells were harvested for protein and probed for phosphorylated and total HER receptors.

Our results from Figure 1 show that pHER2 levels are reduced in both LRes and LTRes cells, suggesting that reactivation of HER2 is not involved in lapatinib resistance in these cells. We therefore further investigated the functional, differential dependence of LRes and TRes on the HER2 and the β1 pathways. To this end, we first subjected LRes cells to trastuzumab and TRes cells to lapatinib, and compared their response to parental cells treated with each agent. As shown in Figure 3A, parental cells were markedly inhibited by both lapatinib and trastuzumab (94.3% and 71.6%, respectively, top panel; also see Table 1). In contrast, LRes cells were only modestly inhibited by trastuzumab (25%, middle panel), an effect that was significantly weaker than the inhibitory effect achieved by trastuzumab in parental cells. TRes cells, on the other hand, were just as sensitive to lapatinib as the parental cells (93.1% and 94.3% growth inhibition, respectively). Collectively these experiments suggest that TRes cells are dependent on the HER2 pathway, while LRes cells are not.

Since HER2 activity is affected by its homo- or hetero-dimerization with EGFR and HER3, we also examined whether a more global differential activation of the HER receptor layer takes place in LRes and TRes cells (Figure 3B). Phosphorylation states of all three receptors--EGFR, HER2, and HER3--were very low in LRes cells which were actively proliferating, suggesting that these cells are driven by a pathway distinct from the HER family. TRes cells, in contrast, had relatively high levels of pEGFR, pHER2, and pHER3 similar to the parental cells, suggesting that the HER pathway is active and explaining why these cells are inhibited by lapatinib (Figure 3A). These data were confirmed with the AU565 model as shown in Additional file 3B.

**Figure 4:**
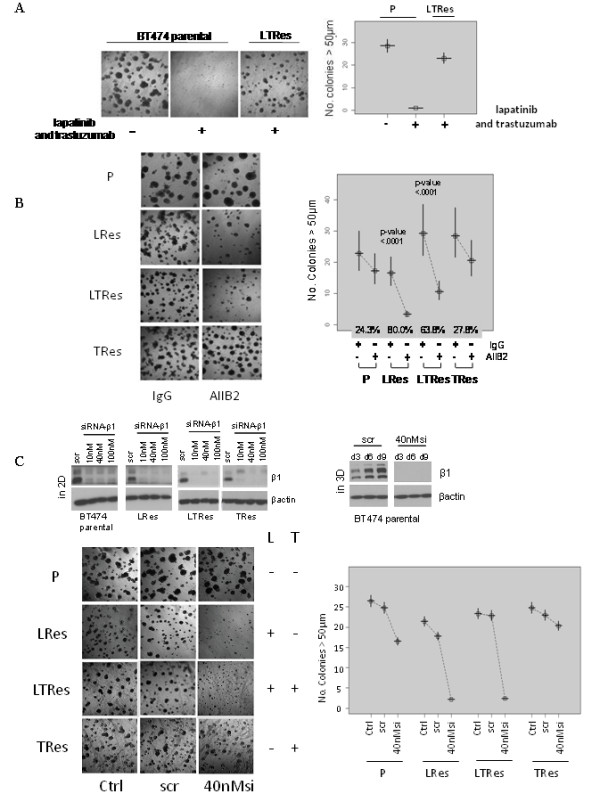
**β1 inhibition impedes colony grow of BT474 LRes and LTRes cells, but not parental or TRes**. (**A**) Parental, LRes, LTRes, and TRes cells were plated in lrECM, subjected to HER2 and/or β1 inhibitors on Day 0, and propagated for 10 to 12 days. (**B**) The Hs_ITGB1_5 siRNA was validated both in 2D and 3D (top), transfected at 40 nMsi into parental, LRes, LTRes, and TRes cells, which were then grown on lrECM for 10 days (bottom).

Having shown that TRes cells are dependent on the HER pathway while LRes cells are not, we next investigated the differential dependence of these resistant clones on β1 integrin. We expanded our studies to include clones resistant to L+T treatment, which our data (Figure 1) suggest are similar to LRes. Upon validating the LTRes phenotype in 3D culture (Figure 4A and Table 1), we assessed the response of LTRes BT474 cells to AIIB2. Similarly to LRes, the LTRes cells also exhibited significant growth inhibition in response to AIIB2 (63.8%, *P *<0.0001, and 80%, *P *<0.0001, respectively, Figure 4B). On the other hand, TRes cells--which retain high HER receptor activity and do not display robust upregulation of β1 integrin signaling (Figure 1)--were only slightly and nonsignificantly inhibited by AIIB2 at a level comparable to parental cells (27.8% and 24.3%, respectively, *P *= .87, Figure 4B).

To further establish the specificity of β1 inhibition, an siRNA approach was employed. β1 protein expression knockdown in 2D as well as in 3D cultures over 9 days was confirmed (Figure 4C, top). The siRNA was then applied in 3D to confirm our findings with AIIB2. As before, siRNA-β1 inhibited parental BT474 cell growth only modestly, but it almost completely inhibited both LRes and LTRes growth (Figure 4C, bottom) and induced apoptosis (Additional file 4). These findings were confirmed with a second independent siRNA sequence (Additional file 1A). Altogether, the β1 inhibition studies using AIIB2 and siRNA-β1 indicate that LRes and LTRes cells are significantly more dependent on the β1 pathway than TRes cells or their parental counterparts, and are thus more sensitive to β1 blockade.

### β1 inhibition overcomes LT resistance in ER-, HER2-amplified HCC1954 cells

**Figure 5:**
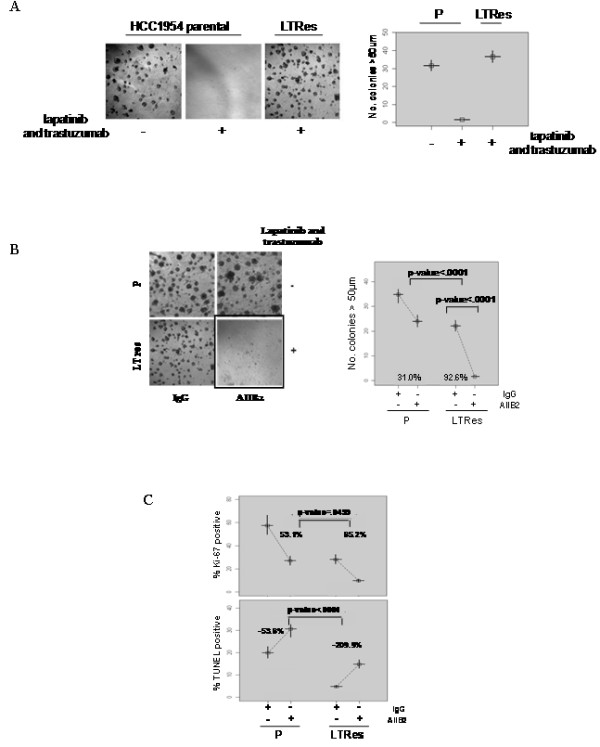
**β1 inhibition overcomes resistance to potent LT therapy in HCC1954 cells**. (**A**) HCC1954 parental and LTRes were plated on lrECM in the presence of LT and assayed for growth response. (**B**) Cells were plated on lrECM, subjected to either AIIB2 or IgG control, and imaged after 12 days. (**C**) Cultures were grown in the presence of appropriate inhibitors for five days and immunostained with Ki67 or TUNEL reagent as before.

The LT combination, which conveys a more complete blockade of the HER receptor layer than HER2-targeted monotherapy, is currently in clinical trials in both the adjuvant and neo-adjuvant settings. To extend our findings in LTRes BT474 cells to another cell line, we chose HCC1954 cells, which are ER-, HER2-amplified, and highly aggressive [39-41], and validated this model on lrECM (Figure 5A and Table 1). Similar to BT474 cells, parental cells were only modestly inhibited by AIIB2 (31.0% reduction in colony number, *P *<0.0001, Figure 5B). In contrast, LTRes cells were almost completely growth-inhibited by blocking β1 integrin (92.6% reduction, *P*-value <.0001, Figure 5B), a reduction significantly greater than parental cells (*P *<0.0001). Examination of an additional ER-, HER2-amplified LTRes cell line--HCC202--corroborated the functional and differential efficacy of AIIB2 on the resistant LTRes derivative (Additional file 3C).

We also examined the effect of β1 blockade on proliferation and apoptosis in HCC1954 cells (Figure 5C). In contrast to BT474 cells, we found that AIIB2 significantly inhibited proliferation in both parental (53.1%, *P *<.0001) and LTRes cells (65.2%, *P *<.0001). Induction of apoptosis, however, was markedly greater (*P *<.0001) in LTRes compared to parental cells (209.9%, *P *<.0001 vs. 53.6%, *P *<.0001, respectively; also see Additional file 4). Thus, although the basal levels of both proliferation and apoptosis varied between parental and LTRes cells, AIIB2 exerted a highly significant differential effect on the induction of apoptosis. These findings indicate that β1 integrin blockade with AIIB2, while reducing 3D colony formation in both BT474 and HCC1954 cell lines, has a predominantly cytostatic effect in BT474 LRes cells but a cytotoxic effect in HCC1954 LTRes cells.

**Figure 6:**
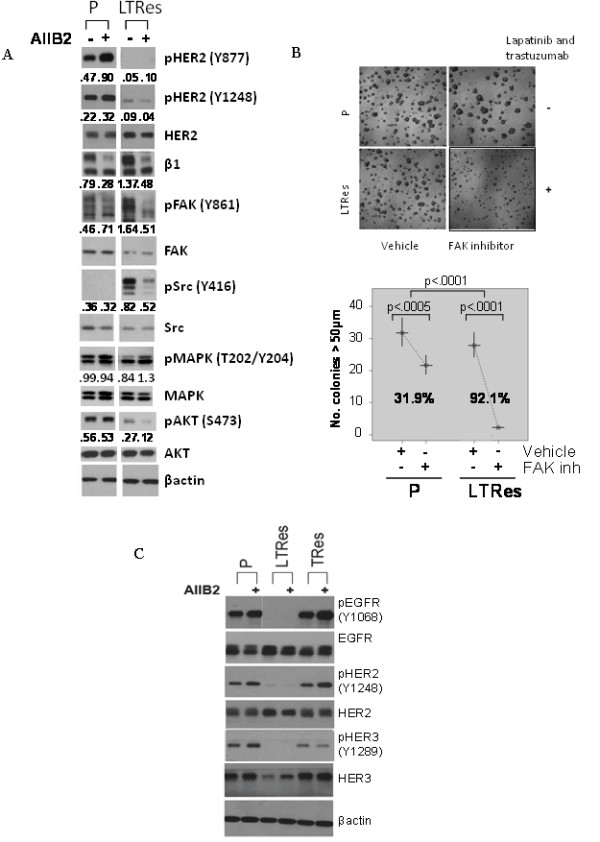
**AIIB2 neutralizes the upregulated pFAK and pSrc expression found in LTRes cells**. (**A**) Parental and LTRes cells were grown on lrECM with AIIB2 or IgG, propagated for five days, and probed for markers. (**B**) The FAK inhibitor was applied to parental and LTRes cells on Day 0 of plating on lrECM and allowed to grow for 12 days. (**C**) 3D cultures were harvested for protein and probed for phospho- and total EGFR, HER2, and HER3.

We next examined how β1 blockade exerted its functional effects on HCC1954 cell growth and survival by surveying β1 signaling intermediates (Figure 6A). Levels of pHER2 at two separate sites were, as in BT474 LRes cells, very low in LTRes cells. Levels of pFAK and pSrc were dramatically higher upon acquisition of resistance to LT therapy, and they were markedly reduced by treatment with AIIB2. AIIB2 affected the levels of β1 and pFAK in parental cells as well, but the significantly weaker functional effect of the β1 inhibitor on 3D culture growth, juxtaposed with its complete growth inhibition of LTRes cells, suggests that β1 signaling plays a more critical role in the growth of LTRes cells where HER2 signaling remains blocked, than it does in parental cells. Finally, pAKT expression is low upon development of LTRes (confirmed in HCC202 cells, Additional file 3D), and it was further decreased by treatment with AIIB2. AKT can be activated by β1 integrin signaling, and this further reduction in pAKT by AIIB2 may also contribute to the growth inhibitory effects in these cells. pMAPK levels, on the other hand, were not inhibited by AIIB2 in HCC1954 LTRes cultures (Figure 6A).

Treatment with the FAK inhibitor PF 573228 confirmed the importance of the β1/FAK signaling axis in HCC1954 LTRes cells (Figure 6B). Inhibition of growth of LTRes cells (92.1%) was significantly greater than in the parental cells (31.9%) (the difference between groups, *P *<0.0001). Similar to our results with AIIB2, these experiments suggest that β1/FAK signaling is critical for the LTRes phenotype.

### TRes HCC1954 cells are only modestly responsive to AIIB2, in contrast to LTRes cells

Similar to BT474 cells, levels of pEGFR, pHER2, and pHER3 in HCC1954 LTRes cells were undetectable in comparison to the high levels found in parental or TRes cells, which are dependent on HER2 for proliferation and survival (Figure 6C). Similar data were also observed in HCC202 cells (Additional file 3D). Interestingly, total HER3 expression was also decreased in HCC1954 LTRes cells, an effect not seen in the BT474 model.

**Figure 7:**
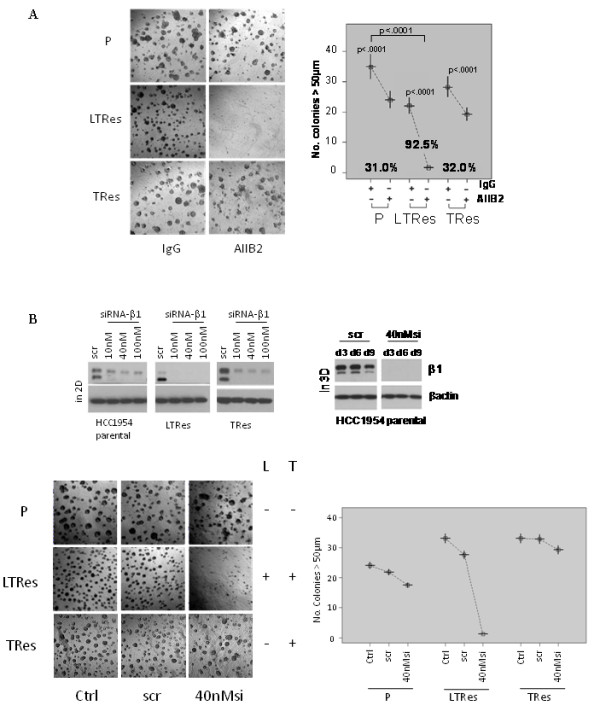
**β1 is critical for the HCC1954 LTRes phenotype, but not parental or TRes**. (**A**) Parental, LTRes, and TRes cells were plated on lrECM, subjected to AIIB2, and allowed to grow for 12 days. (**B**) Cells were subjected to two consecutive rounds of siRNA transfection as before, validated both before and during the experiment (top), and plated in lrECM (bottom).

As above, we wanted to determine the efficacy of the β1 inhibitory antibody in the various HCC1954 derivatives. Of note, we attempted to assay LRes HCC1954 cells in 3D but could not grow them on lrECM. We thus reassessed parental HCC1954 cells alongside the LTRes and long term-cultured TRes derivative cells (Table 1) in 3D, where HCC1954 cells are *de novo *resistant to trastuzumab due to a mutation in PI3K. We found that TRes, like parental cells, were only modestly but significantly responsive to AIIB2 (32.0% inhibition, *P *<.0001 and 31.0%, *P *<.0001, respectively), whereas LTRes cells, as above, were 92.5% inhibited (*P *<.0001, Figure 7A). The degree of inhibition between parental and TRes cells was not significant (*P *= .52) but was indeed significant between parental and LTRes cells (*P *<.0001).

Using a genetic approach, we confirmed knockdown of β1 protein levels post transfection with siRNA-β1 or a scrambled sequence, before plating on lrECM (Figure 7B, top). Cultures were imaged after 10 days (Figure 7B, bottom) and protein extracted every 3 days to confirm sustained β1 knockdown (Figure 7B, top). A second independent sequence (Additional file 1B) yielded similar results. siRNA-β1 inhibited growth of the parental and TRes cells only modestly, but inhibited LTRes cell growth completely (Figure 7B). Collectively these results suggest that HCC1954 LTRes cells are more dependent on β1 signaling than either the parental or TRes cells, and confirm with a second cell model that resistance to lapatinib-containing regimens--where HER2 remains inhibited--is exceedingly more responsive to β1 inhibition than in TRes, where HER signaling remains active.

## Discussion

Multiple mechanisms of trastuzumab resistance have been proposed in both preclinical and clinical studies [42], such as incomplete blockade of the HER receptor layer [36,37,43], PTEN loss, or activating mutations in PI3K, but the causes of acquired resistance to lapatinib are less clear [9]. The microenvironment has been shown to influence various cell survival and proliferation pathways [33,44,45]. Here, we provide evidence that the β1 integrin, a receptor that transmits signals from the microenvironment, plays an important role as an escape pathway in acquired resistance to L and LT, where HER signaling remains strongly inhibited.

We now demonstrate that HER2-overexpressing LRes and LTRes cells, while maintaining strong inhibition of phosphorylation of EGFR, HER2, and HER3, exhibit marked up-regulation of β1 signaling by activation of its downstream kinases, FAK and Src. Enhancement of pFAK and pSrc levels is specifically greater in LRes and LTRes than in TRes cells. Several studies independently investigating mechanisms of resistance to L and T have shown that LRes is associated with continued inhibition of HER receptors [46] or the HER pathway [12,47], while TRes is associated with reactivation of the HER pathway [48,49]. We found β1 signaling components to be more prominently up-regulated in L- and LTRes cells compared to TRes cells. As in the prior studies, LRes and LTRes cells have low levels of phosphorylated HER2, EGFR, and HER3, indicating that the HER pathway is still shut down.

Using multiple HER2-amplified cell line models, some ER+ and some ER-, and their therapeutically resistant derivatives grown in lrECM, we found that parental cells were only modestly responsive to β1 inhibition by the antibody AIIB2, by siRNA-β1, or by FAK inhibition with PF573228. LRes and LTRes cells, in contrast, were significantly growth-inhibited, suggesting a greater dependence of these resistant cells on β1 signaling than their parental counterparts. In further elucidating the mechanism of action underlying β1 inhibition, we found that AIIB2 modulated β1 expression and effectively suppressed both pFAK and pSrc, as well as pAKT, in both parental and LRes or LTRes derivatives. It is possible that the β1 pathway also promotes growth and survival in parental cells, but our data suggest that HER2 remains the primary driver (Figure 3A). It is interesting to note that while up-regulation of β1 protein in resistant cells was not always observed (Figures 1, 2D), β1 pathway activation could potentially be achieved several ways, which include the release of ECM ligands, integrin clustering, and/or the activation of downstream markers. Our studies showed that LRes and LTRes cells exhibit increases in the β1 downstream markers pFAK and pSrc when compared to the parental cells, and that inhibition of β1 is able to reduce the high levels of phosphorylated FAK and Src found in cells resistant to lapatinib. These observations suggest that some HER2-overexpressing breast cancer cells become more dependent on β1 signaling as they acquire resistance to L, a potent HER receptor inhibitor, and that blocking this escape pathway can restore inhibition of tumor growth.

Interestingly, we found that in cells resistant to L-containing regimens, blocking β1 can elicit an apoptotic or predominantly anti-proliferative response, depending on the cell line studied. The molecular mechanisms responsible for this differential response to β1 inhibition are not clear, although it is interesting to note that the inhibition of BT474 cell proliferation coincides with a reduction in pMAPK, while inhibition of HCC1954 LTRes cells is associated with increased apoptosis and a marked reduction in pAKT.

In comparing acquired LRes or LTRes to single-agent TRes, we found that β1 inhibition by AIIB2 was significantly and specifically effective in those cells resistant to lapatinib-containing treatments. Importantly, even doubling the dose of lapatinib in LRes and LTRes cells did not inhibit growth of either cell line in 3D (Additional File 5), further suggesting that growth of these L-resistant lines is independent of HER2. Under these conditions, our data suggest that the β1 pathway compensates at least in part for the blockade of HER signaling. Both acquired (BT474) and *de novo *(HCC1954) TRes cells, on the other hand, maintain their dependence on HER2, as evidenced by their sensitivity to lapatinib [13,48] (Figure 3A) and the high levels of pEGFR, pHER2, and pHER3 (Figures 3B, 6C).

There have been recent publications suggesting a role for β1 integrin in intrinsic trastuzumab resistance [34,44]. Our studies focused on *acquired *resistant cell lines developed through chronic exposure. As such, our data do not dispute these reports. We also find that growth of TRes cells is modestly inhibited by blocking β1. Our results suggest, however, that β1 integrin signaling is a much more prominent escape pathway for HER2-amplified tumors treated by L or LT, than by T alone. Studies of tumor tissue from patients with acquired resistance to L or T are required to learn whether these preclinical observations have clinical relevance.

## Conclusions

Although multiple mechanisms likely underlie and contribute to lapatinib resistance, our data suggest that β1 integrin signaling is a promising therapeutic target to block and thereby inhibit growth of resistant tumors in patients.

## Abbreviations

EGFR: Epidermal Growth Factor Receptor; ER: Estrogen Receptor; T: trastuzumab; L: lapatinib; lrECM: laminin-rich extracellular matrix; p: phosphorylated; Res: resistant

## Competing interests

The authors declare that they have no competing interests.

## Authors' contributions

CH conducted the molecular studies presented in this manuscript, including 3D culturing, application of pharmacological and genetic approaches to inhibit β1, immunostaining, protein extraction, and immunoblotting. CP, MR, and MJB contributed substantially to the conception and design of the data. SH performed all statistical analyses necessary for each molecular study. RW and YW developed the lapatinib, trastuzumab, and LT resistant cell lines through long term exposure of parental cells to each inhibitor or set of inhibitors. CKO and RS conceived of the study and its design and implementation. All authors read and approved the final manuscript.

## Supplementary Material

Additional file 1 **A second siRNA sequence applied to BT474 and HCC1954 cells in 3D lrECM confirms that LRes and LTRes cells depend more critically on β1 than their parental counterparts**. Double, consecutive rounds of siRNA transfection at 40 nM were executed and cells plated directly onto lrECM for 10 days, followed by quantification. Click here for file

Additional file 2 **Phosphorylated levels of the β1 downstream kinases FAK and Src are increased in additional HER2-overexpressing cell line models upon acquisition of resistance to lapatinib (L)-containing HER-targeted therapies**. (**A**) Parental (P) AU565 and (**B**) HCC202 cells resistant to lapatinib (LRes) and combination (LTRes) treatment strategies were developed by long-term exposure in 2D. Protein extracts were probed for β1, pHER2, pFAK, and pSrc, as well as totals. Click here for file

Additional file 3 **β1 blockade overcomes resistance to lapatinib-containing regimens in AU565 and HCC202 cells and abrogates upregulated pFAK and pSrc expression**. (**A**) and (**C**) Cells were propagated in 3D lrECM and treated with respective inhibitors and/or AIIB2. Statistical analysis was conducted as in Figure 2. (**B **and **D**, left) 3D extracts of AU565 cells exhibit upregulated protein expression of β1, pFAK, and pSrc upon acquisition of resistance to lapatinib. These effects are neutralized upon application of the β1 inhibitory antibody AIIB2. Expression of phosphorylated levels of MAPK and AKT are decreased in LRes cells in comparison to their parental counterparts. (**B **and **D**, right) The HER receptor layer is effectively inhibited in L- and LT-Res cells but remains active in both parental and TRes cells. Click here for file

Additional file 4 **Genetic blockade of β1 by siRNA in BT474 and HCC1954 cells induces apoptosis**. Cells were transfected with siRNA, plated onto lrECM, propagated for five days, then harvested using the TUNEL assay as in Figure 2C. Click here for file

Additional file 5 **Doubling the dose of lapatinib in cells resistant to lapatinib-containing regimens does not dramatically affect growth**. BT474 LRes and HCC1954 LTRes cells were first primed in 2D with 2 μM lapatinib (twice the usual dose) for five days. Cells were then plated onto lrECM, propagated for 12 days, and quantified. Click here for file
