# Supplementary figures and images for: β1 integrin mediates an alternative survival pathway in breast cancer cells resistant to lapatinib
Source: Breast Cancer Res. 2011 Aug 31;13(4):R84. doi: 10.1186/bcr2936 (PMC3236347; doi:10.1186/bcr2936)

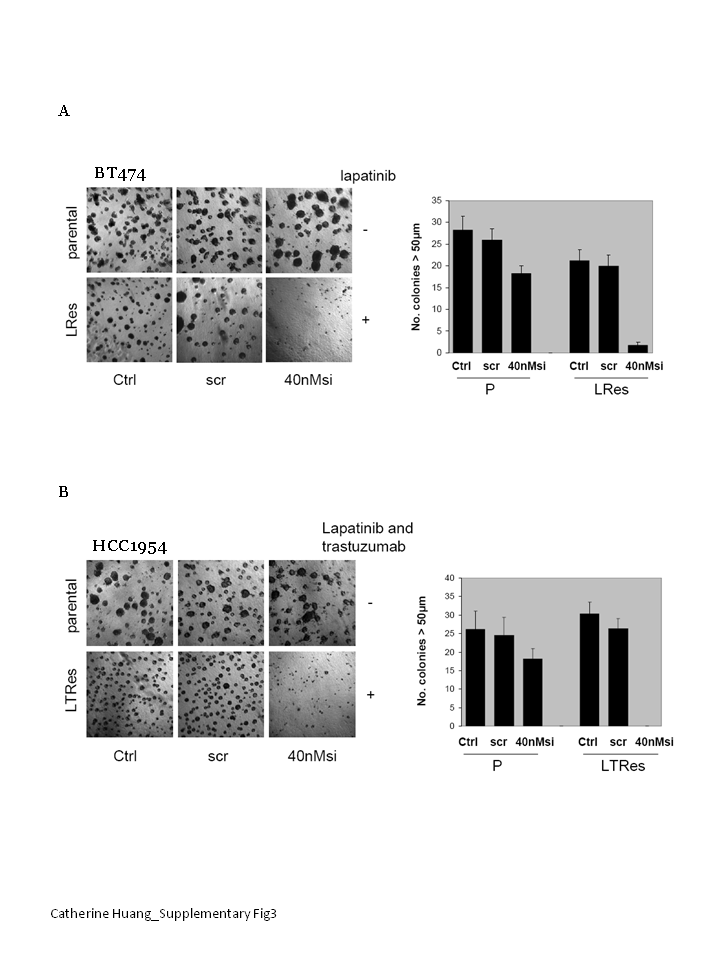

Supplement: Additional file 1 — A second siRNA sequence applied to BT474 and HCC1954 cells in 3D lrECM confirms that LRes and LTRes cells depend more critically on β1 than their parental counterparts. Double, consecutive rounds of siRNA transfection at 40 nM were executed and cells plated directly onto lrECM for 10 days, followed by quantification. [file bcr2936-S1.TIFF]

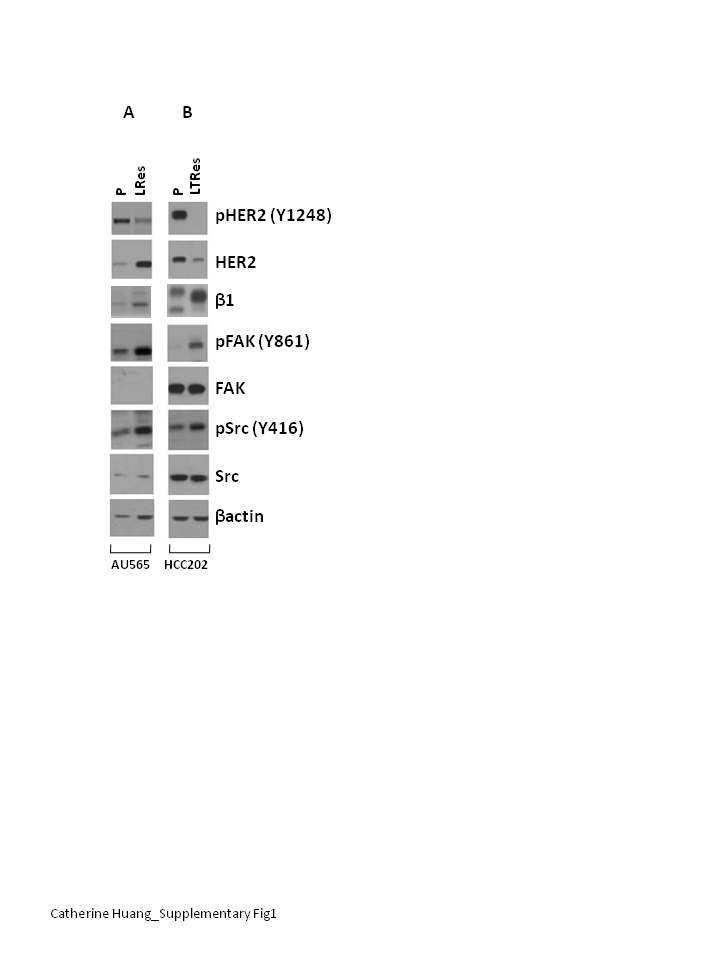

Supplement: Additional file 2 — Phosphorylated levels of the β1 downstream kinases FAK and Src are increased in additional HER2-overexpressing cell line models upon acquisition of resistance to lapatinib (L)-containing HER-targeted therapies. (A) Parental (P) AU565 and (B) HCC202 cells resistant to lapatinib (LRes) and combination (LTRes) treatment strategies were developed by long-term exposure in 2D. Protein extracts were probed for β1, pHER2, pFAK, and pSrc, as well as totals. [file bcr2936-S2.TIFF]

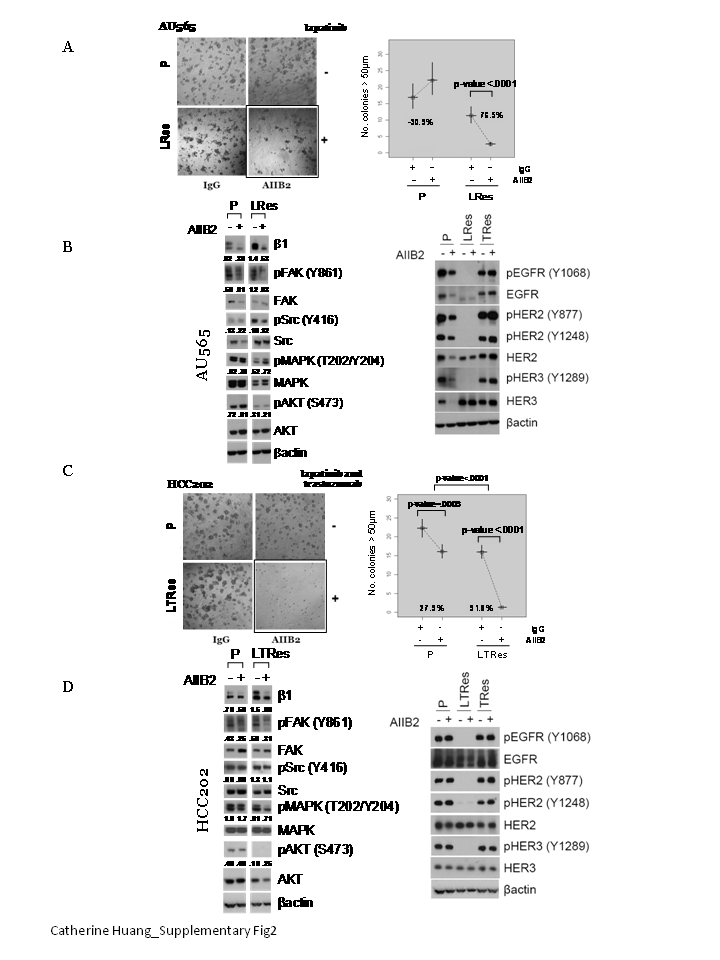

Supplement: Additional file 3 — β1 blockade overcomes resistance to lapatinib-containing regimens in AU565 and HCC202 cells and abrogates upregulated pFAK and pSrc expression. (A) and (C) Cells were propagated in 3D lrECM and treated with respective inhibitors and/or AIIB2. Statistical analysis was conducted as in Figure 2. (B and D, left) 3D extracts of AU565 cells exhibit upregulated protein expression of β1, pFAK, and pSrc upon acquisition of resistance to lapatinib. These effects are neutralized upon application of the β1 inhibitory antibody AIIB2. Expression of phosphorylated levels of MAPK and AKT are decreased in LRes cells in comparison to their parental counterparts. (B and D, right) The HER receptor layer is effectively inhibited in L- and LT-Res cells but remains active in both parental and TRes cells. [file bcr2936-S3.TIFF]

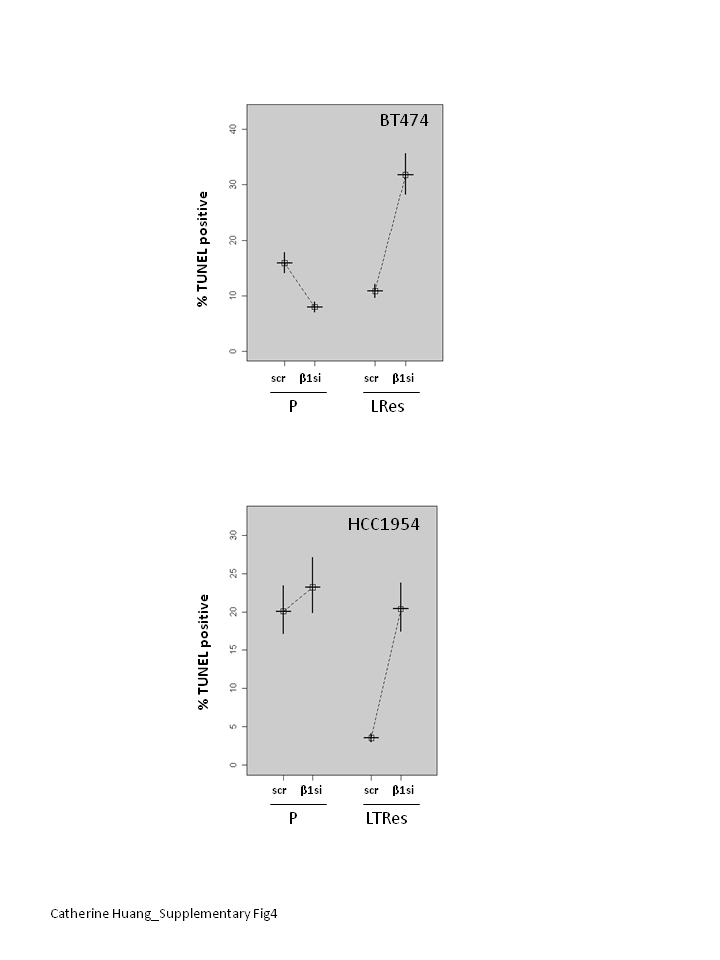

Supplement: Additional file 4 — Genetic blockade of β1 by siRNA in BT474 and HCC1954 cells induces apoptosis. Cells were transfected with siRNA, plated onto lrECM, propagated for five days, then harvested using the TUNEL assay as in Figure 2C. [file bcr2936-S4.TIFF]

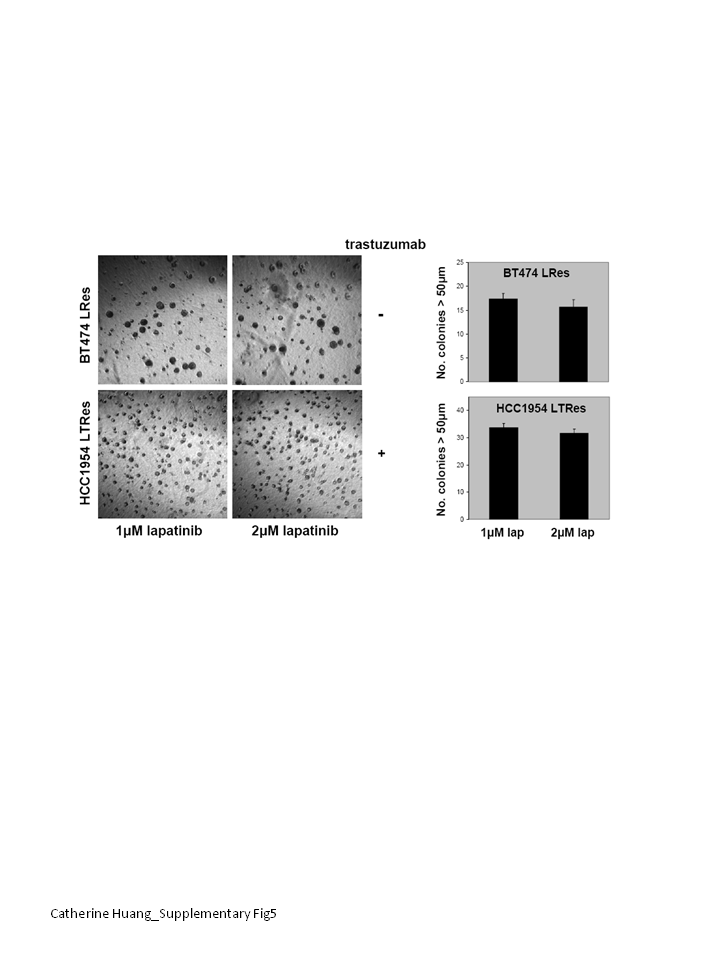

Supplement: Additional file 5 — Doubling the dose of lapatinib in cells resistant to lapatinib-containing regimens does not dramatically affect growth. BT474 LRes and HCC1954 LTRes cells were first primed in 2D with 2 μM lapatinib (twice the usual dose) for five days. Cells were then plated onto lrECM, propagated for 12 days, and quantified. [file bcr2936-S5.TIFF]
